# Throat carriage rate, associated factors, and antimicrobial susceptibility pattern of group A *Streptococcus* among healthy school children in Jigjiga City, Eastern Ethiopia

**DOI:** 10.1186/s12887-022-03294-2

**Published:** 2022-04-26

**Authors:** Shamil Barsenga, Habtamu Mitiku, Tewodros Tesfa, Tadesse Shume

**Affiliations:** 1 Department of Medical Laboratory Sciences, Jigjiga University, College of Medicine and Health Sciences, Jigjiga, Ethiopia; 2 Department of Medical Laboratory Sciences, Haramaya University, College of Health and Medicine Sciences, P.O.Box- 235, Harar, Ethiopia

**Keywords:** Throat carriage, Group A *Streptococcus*, Antimicrobial susceptibility pattern, Children, Jigjiga Ethiopia

## Abstract

**Background:**

Group A *Streptococcus* has been recognized as an important human pathogen and it remains among the top ten causes of mortality from an infectious disease. Group A *Streptococcus* throat carriage plays an important role in the development of infection and transmission to contacts. In Ethiopia, there is little information about screening of children for group A *Streptococcus* carriage.

**Objective:**

This study was aimed to assess the magnitude of throat carriage, associated factors, and antimicrobial susceptibility pattern of group A *Streptococcus* among healthy school children in Jigjiga city, Eastern Ethiopia from 12 April to 27 May 2021.

**Method:**

A cross-sectional study was conducted enrolled by simple random sampling. Data on socio-demographic and related characteristics were gathered using pretested structured questionnaire. The throat sample was collected from 462 healthy school children and immediately transported to Jigjiga University Sultan Sheik Hassan referral hospital laboratory for investigation. Identification of group A *Streptococcus* was done by colony characterstics, gram staining, catalase negativity, bacitracin sensitivity, and Pyrrolidonyl arylamidase tests. Antibiotic susceptibility test was done on Muller-Hinton agar containing 5% sheep blood by modified Kirby-Bauer disc diffusion method. The data were coded, cleaned, and entered onto EpiData Version 3.1 then exported to SPSS version 26.0 for analysis. Bivariate and multivariable logistic regression through adjusted odds ratio (AOR) was used to determine the relationship between culture-positivity rates of GAS and predictor variables. A *p*-value < 0.05 was taken as statistically significant on multivariable analysis.

**Results:**

The overall prevalence of group A *Streptococcus* throat culture rate was 10.6% (95%CI; 8.1%—13.7%). Previous family member who had a sore throat, children living with larger families (more than 11 members), and children living with non-immediate families were significantly associated with culture-positivity rates of GAS. Children who live with a family member with a sore throat compared with those who lived with in a family with no history of sore throat (AOR = 2.51; 95%CI 1.09–5.73), children who live with a large family comared to children living in families with less members (AOR = 4.64; 95% CI 1.53–14.1), and children who live with non-immediate families compared to children living with their mothers (AOR = 3.65; 95% CI 1.39 – 9.61), showed significant association with group A *Streptococcus* carriage rate. Resistance to all other antibiotics tested was low (< 5%). Multidrug resistance was found in 4.1% of isolates.

**Conclusion:**

The present study showed 10.6% throat carriage of group A *Streptococcus*. Family member with a sore throat, having a large family, and living with non-immediate families have all been identified as independent predictors of carriage prevalence.

## Introduction

Group A *Streptococcus* (GAS) has been an important human pathogen since the early days of modern microbiology, and it is still among the top ten causes of mortality from an infectious disease [1]. All diseases caused by GAS are most common in settings of poverty, where living conditions promote transmission of the organism, and prevention and treatment programs are less likely to be present or effective [2].

Group A *Streptococcus* can infect people of any age, however children are more likely to be infected [3]. Group A *Streptococcus* is responsible for a wide range of clinical Syndrome, including impetigo and pharyngitis, as well as more serious disorders such as streptococcal toxic shock syndrome (STSS), meningitis, pneumonia, or cellulitis etc. Furthermore, autoimmune diseases such as acute rheumatic fever (ARF) can be triggered by recurrent episodes of GAS infection [4].

According to global disease burden figures in 2005, WHO ranked GAS as the ninth leading cause of human mortality, with the majority of deaths attributable to invasive GAS infections and rheumatic heart disease (RHD) [2]. The prevalence of GAS disease is estimated at > 18.1 million cases with an incidence of > 1.78 million cases per year [1, 5]. The most common infection caused by GAS is pharyngitis in children between 5 and 15 years of age [6]. It is responsible for approximately 15–30% of cases of pharyngitis in children [7]. Failure to eradicate streptococci from the pharynx occurs in about one-third of non-treated cases, giving rise to the carrier status in those individuals [8]. Untreated GAS pharyngitis may trigger ARF and its sequela, RHD, remain important public health problems in low and middle-income countries [1, 9].

The existence of the carrier state is reported to be as high as 15–20% in previous studies [10]. Carriers of GAS may represent a potential source for the acquisition of infections for other children and adults [11, 12]. According to a review article published by Oliver and colleagues in 2018, reported 10.5% and 5.9% pooled prevalence of GAS carriage in children from high-income countries and children from low/middle-income countries, respectively [13]. A few studies have been done in Africa on the GAS carriage which ranged around 9.0% [8].

Crowding, limited access to hygiene, inadequate medical care, housing quantity and quality, healthcare access and quality, education, or economic advantage are all risk factors for GAS infection or colonization (carriage) [1].

Even though GAS causes significant problems, there is scarcity of information in Ethiopia [14, 15] and only a few investigations on GAS have been conducted in previous years [14], particularly it was untouched in Jigjiga, Eastern Ethiopia. Therefore, this study was designed to assess throat carriage prevalence, associated factors, and antimicrobial susceptibility pattern of GAS among healthy school children in Jigjiga city, Eastern Ethiopia.

## Methods and materials

### Study area and period

The study was conducted in Jigjiga, a city in the Somali Region of Ethiopia from 12 April to 27 May 2021. Jigjiga is 630 km east far from the capital city, Addis Ababa. It is estimated that 763,509 people live in Jigjiga. There were ninety-nine (99) primary schools in the city of Jigjiga. Of which, 74 private schools, and 25 government schools. A total of 36,507 students are present. Among these, 20,056 were males and 16,451 were females [16].

### Study design and population

A cross-sectional study was conducted. School children from governmental and private primary schools who were randomly selected from twenty schools using a lottery system. Written informed consent was obtained from their parents for children aged less than 11 years. Furthermore, children aged 11 years and above were insisted to give a written assent.

### Inclusion and exclusion criteria

All students in selected schools aged 7–14 years old who attend the class during the study period were included in the study. All children on antibiotics for the previous two weeks and children with throat infection or any related sign and symptoms of pharyngitis were excluded from the study.

### Sample size determination

A single proportion formula was used to determine the sample size for this quantitative study by considering the following assumptions**:** a prevalence of asymptomatic pharyngeal carriage rate of GAS among healthy school children in Hawassa, southern Ethiopia with a prevalence of 12.2% [15], 95% confidence level, and a margin of allowable error of 5%,. To minimize errors arising from the likelihood of non-compliance, 10% of the sample size was added. The final sample size was 178.

On the other hand, sample size was determined by considering different factor-associated GAS colonization using double population proportion formula with the assumption of a two-sided confidence level of 95%, the margin of error 5%, and power of 80%. Finally, 231 study participants were calculated. So, the sample size for a single population proportion was smaller than the sample size calculated for the second calculated double population proportion, which is a sample size of 231 was used. Because we used a multi-stage sampling method, we introduced a design effect. As a result, 231 was multiplied by 2, resulting a total sample size of 462.

### Sampling procedure and sampling technique

From a total of 99 primary schools, twenty (20) schools were chosen at random through a lottery system, from government and private schools. Then the determined sample size for the study was proportionally allocated to each selected school. A simple random sampling method was used to enroll children. In order to include study participants in the study, a multistage sampling technique was used. Samples were taken from children whose parents agree to participate until the sample size attend from each selected school.

### Sample collection and transportation methods

Data on the socio-demographic characteristics of the parents/guardians and children, as well as the children's clinical history, were collected using a structured questionnaire adopted from previous studies [15, 17]. Two professional nurses administered the questionnaires, and two trained laboratory personnel used cotton swabs to collect a throat sample from a selected child. The throat swab samples were placed in Amie's transport media. Within two hours, the sample was sent in a cold chain to Jigjiga University Sultan Sheik Hassan referral hospital laboratory for investigation.

### Laboratory investigation

The throat sample was cultured on 5% sheep blood agar plates (Himedia, India) by rolling the swab over a small area of the plate and streaking the sample with a sterile loop, then incubated at 37 °C with 5% CO_2_ atmosphere for 24 h. A catalase test and gram staining were performed on colonies having ß-hemolysis. All catalase-negative and gram-positive cocci were subcultured for 24 h at 37 °C on 5% fresh blood agar plates with a Bacitracin disk in a 5% CO_2_ atmosphere to differentiate colonies suspected to be *S. pyogenes*. Any zone of inhibition around the bacitracin disk was a candidate for Pyrrolidonyl arylamidase (PYR) tests, change of color to red /purple was confirmed culture positive for GAS. [18, 19].

### Antimicrobial susceptibility testing

The drug susceptibility test was done by a disk diffusion method by using Muller Hinton Agar (MHA) supplemented with 5% sheep’s blood. Colony suspension was made using normal saline (0.85% NaCl) equivalent to 0.5% McFarland standard from grown overnight colonies (18–24 h) on sheep blood agar plate. The suspension was inoculated to an MHA plate with 5% sheep’s blood using a culture swab and incubated at 5% CO_2_ for 18 to 24 h. Drug disks containing penicillin (10 IU), erythromycin (15 g), azythromycin (15 g), amoxicillin (10 g), chloramphenicol (30 g), ceftriaxone (30 g), vancomycin (30 g), and tetracycline (10 g) were utilized. The drugs are selected in accordance with the Ethiopian Drug Administration standard treatment guidelines for health centers and Control Authority's and the Clinical Laboratory Standards Institute's (CLSI). Finaly, the zone of inhibition was measured with a ruler, then recorded and compared to the Clinical and Laboratory Standards Institute [20].

### Data quality control

The questionnaire was written in English, then translated into Amharic and Somali, and finally back to English to ensure uniformity. The questionnaire was pre-tested before actual data collection begins. For laboratory testing, the sterility of each batch of produced media was determined by incubating 5% of the culture media in a 5% CO_2_ enriched atmosphere at 37 °C for 24 h before using it. *Streptococcus pyogenes* (ATCC 12,696) and *Streptococcus agalactae* (ATCC 13,813) were used as a positive and negative control respectively. Quality assurance in antimicrobial susceptibility was done by repeating the selected tests on the same day as the original.

### Method of data analysis

The data was entered and coded into the Epi-Data version 3.1 upon creating the questionnaire template. The entered data was cleaned to ensure the validity of all recorded data. The analysis was then carried out using SPSS version 26.0. Descriptive statistics and frequency tables were used to summarize the data. The magnitude of the association between the different variables with the throat culture positive for GAS was measured by the adjusted odds ratio (AOR) with a 95% confidence interval. Bivariate and multivariable logistic regression analysis was made to obtain the odds ratio and the confidence interval of statistical associations. All variables that are significant at *p*-value < 0.25 in the bivariate analysis were considered for multivarible analysis. Previous family member who had a sore throat, children living with larger families (more than 11 members), and children living with non-immediate families were adjusted and calculated through adjusted odds ratios to measured the strength of statistical association at 95% confidence intervals and statistical significance was declared at *p* < 0.05.

## Results

### Sociodemographic characteristics of study participants

A total of 462 schoolchildren were participated in the study with a response rate of 100%. About 244 (52.8%) of study participants were females. Two hundred seven students (57.8%) were from governmental primary schools, while 195 (42.2%) were from a private primary school in Jigjiga city administration. The study participants' ages range from 7 to 14, with a mean age was of 10.9 years (SD ± 1.89). The majority (89.4%) of the participants live in a city. Two hundred twenty-nine (49.6%) of research participants lived with 6 to 10 family members, while 214 (46.3%) shared a sleeping room with 1 to 3 people. A large percentage of (73.5%) the parents/guardians of the children were married. Mothers were the primary caregivers in 320 (69.3%) of the cases, and 331 (71.6%) of the respondents had acquired formal education. One hundred forty-three (30.9%) of the respondents/caregivers were merchants and 108 (23.4%) were government employees (Table 1).

**Table 1:** Sociodemographic characteristics of children and parent/guardians who participated in a study of group A *Streptococcus* throat carriage prevalence study—Jigjiga city, Eastern Ethiopia, April 12 to May 27, 2021

Variables		Frequency	Percentage (%)
**Socio-demographic characteristics of the child**			
Age of the child in years	7–10	199	43.1
	11–14	263	56.9
Sex of the child	Male	218	47.2
	Female	244	52.8
Residence	In the town	41 3	89.4
	Outskirt	49	10.6
Family Size (number of person)	1–5	142	30.7
	6–10	229	49.6
	11–15	91	19.7
Sharing a sleeping Room	1–3	214	46.3
	4–6	199	43.1
	7–9	49	10.6
**The social condition of the parent/guardian of the child**			
Marital status	Married	340	73.5
	Divorced	35	7.6
	Widowed	17	3.7
	Single	70	15.2
Care givers	Mother	320	69.3
	Father	109	23.6
	Non-immediate family^a^	33	7.1
Educational status	Unable to read and write	131	28.4
	Received formal education^b^	331	71.6
Occupation	Searching for Job	25	5.4
	Stay home parent	106	22.9
	Merchant	143	31.0
	Private employee	80	17.3
	Government employee	108	23.4
Monthly income (ETB)	250 – 1000	108	23.4
	1000 -3000	56	28.6
	> 3000	222	48.1

^a^Grandparent, aunt, and/or uncle

^b^Primary (1–8), secondary (9–12), tertiary (diploma and above), and/or degree and above

*ETB* Ethiopian Birr

### Clinical history of the study participants

The majority (76%) of the study participants had no history of hospitalization in the previous 5 years and 87.9% of family member had no history sore throat in the last 30 days. Nearly, half of the parents 47.6% claimed that they had never given antibiotics to their children.

### Prevalence of group A *Streptococcus*

Among 462 in healthy school children, 49 (10.6%) (95% CL; 8.1%—13.7%) were confirmed to have GAS in their throats.

## Analysis for factors associated with GAS colonization

In bivariate regression analysis; caregivers, monthly income, family size (number of person), sharing a sleeping room, Sore throat (within the last 30 days), and previous family member who had a sore throat showed a significant association at a *p*-value of < 0.25 and were considered as a candidate for multivariate logistic regression. Through a multivariable analysis: previous family member who had a sore throat, children living with larger families (more than 11 members), and children living with non-immediate families were significantly associated with culture-positivity rates of GAS at *p*-value < 0.05 after adjusted for confounding factors.

Children who lived with a family member who had a sore throat previously were twice likely to have GAS in their throat compared with those who lived with no family member who had a sore throat (AOR = 2.51; 95%CI 1.09–5.73;). Children living in families with more than 11 members were four times more likely carrying GAS compared to children living in families with less members (AOR = 4.64; 95% CI 1.53–14.1). Children living with non-immediate families were more than three times more likely to have GAS compared to children living with their mothers (AOR = 3.65;95%; CI: 1.39 – 9.61) (Table 2).

**Table 2:** Bivariate and multivariable analysis of sociodemographic and clinical variables associated with group A *Streptococcus* throat carriage among healthy school children—Jigjiga city, Eastern Ethiopia, April 12 to May 27, 2021

Variables		Culture result		COR (95% C. I)	*p*-value (a)	AOR (95% C.I)	*p*-value(b)
		**GAS negative *n* = 413 (89.4%)**	**GAS positive *n* = 49 (10.6%)**				
**Socio-demographic characteristics**							
Age of the child	7–10	181(91.0)	18(9.0)	0.74 (0.40 -1.37)	0.35		
	11–14	232 (88.2)	31 (11.8)	1			
Sex of the child	Male	197(90.4)	21 (9.6)	1			
	Female	216 (88.5)	28 (11.5)	1.22 (0.67 – 2.21)	0.52		
**Parental/Guardian Social condition**							
Marital Status	Single	63 (90)	7 (10)	0.97 (0.41 – 2.28)	0.94		
	Widowed	13 (76.4)	4 (23.5)	2.68 (0.83 – 8.67)	0.10		
	Divorced	32 (91.4)	3 (8.6)	0.82 (0.24 – 2.80)	0.75		
	Married	305 (89.7)	35 (10.3)	1			
Caregivers	Non-immediate family^a^	24 (72.7)	9 (27.3)	3.15 (1.36 – 7.34)	0.008*	3.65 (1.39 -9.61)	0.009**
	Father	104 (95.4)	5 (4.6)	0.49 (0.20 – 1.20)	0.12*	0.50 (0.18 – 1.36)	0.174
	Mother	285 (89.1)	35 (10.9)	1		1	
Education status	Unable to read and write	111 (84.7)	20 (15.3)	1.88 (1.03 – 3.45)	0.043*	1.11 (0.53 – 2.30)	0.78
	Recived formal education^b^	302 (91.2)	29 (8.8)	1			
Occupation	Searching for Job	23(92.0)	2 (8.0)	0.69 (0.15 – 3.33)	0.65		
	Stay home parent	88 (83.0)	18 (17.0)	1.64 (0.75– 3.59)	0.22		
	Merchant	129 (90.2)	14 (9.8)	0.87 (0.38 – 1.96)	0.73		
	Private employee	77 (96.3)	3 (3.8)	0.31 (0.08 – 1.14)	0.08		
	Government employee	96 (88.9)	12 (11.1)	1			
Monthly income (ETB)	250 – 1000	86 (79.6)	22 (20.4)	3.08 (1.56 – 6.09)	0.001*	1.87 (0.85 – 4.15)	0.21
	1000 – 3000	122 (92.4)	10 (7.6)	0.98 (0.44 – 2.23)	0.99	0.79 (0.33 – 1.91)	0.61
	> 3000	205 (92.3)	17 (7.7)	1		1	
**Child living condition**							
Family size	1 – 5	134 (94.4)	8 (5.6)	1		1	
	6- 10	211 (92.1)	18 (7.9)	1.43 (0.60 – 3.38)	0.42	1.41 (0.53 – 3.72)	0.493
	11 – 15	68 (74.7)	23 (25.3)	5.67 (2.40 – 13.3)	0.000*	4.64 (1.53 – 14.1)	0.007**
sharing a sleeping Room	1 – 3	201 (93.9)	13 (6.1)	1		1	
	4 – 6	174 (87.4)	25 (12.6)	2.22 (1.10 – 4.48)	0.025*	1.48 (0.64 – 3.41)	0.356
	7 – 9	38 (87.8)	11(22.4)	4.48 (1.87 – 10.7)	0.01*	1.89 (0.59 – 6.06)	0.280
Residence	In the town	372 (90.1)	41 (9.9)	1			
	Outskirt	41 (83.7)	8 (16.3)	1.77 (0.78—4.03)	0.174		
Duration of child stay in school	Half-day	375 (89.3)	45 (10.7)	1			
	Full day	38 (90.5)	4 (9.5)	1.14 (0.39 – 3.34)	0.811		
**Clinical History of the child**							
Earlier antibiotic use	1 – 6 months	61 (89.7)	7 (10.3)	1			
	6 – 12 months	73 (90.1)	8 (9.9)	0.84 (0.37 – 1.87)	0.662		
	> 12 month	84 (90.3)	9 (9.7)	0.86 (0.37 – 1.98)	0.714		
	No	195 (88.6)	25 (11.4)	0.89 (0.37 – 2.17)	0.806		
Hospitalization history (< 5 years)	Yes	96 (86.5)	15 (13.5)	1.46 (0.76 – 2.79)	0.256		
	No	317 (90.3)	34 (9.7)	1		1	
Previous family member with a sore throat (the last 30 days)	Yes	45 (80.4)	11 (19.6)	2.37 (1.13 – 4.95)	0.022*	2.51 (1.09 – 5.73)	0.029**
	No	368(90.6)	38 (9.4)	1		1	
Sore throat (within the last 30 days)	Yes	79 (83.2)	16 (16.8)	2.05 (1.08 – 3.91)	0.03*	1.86 (0.90 – 3.85)	0.093
	No	334 (91.1)	33 (8.9)	1			

^*^ = *p* value < 0.25; ** = Statistically significant (*p* < 0.05)

^a^Grandparent, aunt, and/or uncle

^b^Primary (1–8), secondary (9–12), tertiary (diploma and above), and/or degree and above

*AOR* Adjusted Odds Ratio, *COR* Crude Odds Ratio

### Antibiotic susceptibility testing

To determine the antimicrobial susceptibility patterns of isolates, those 49 bacterial isolates were tested for eight different antimicrobials. Majority of them were susceptible to amoxicillin 44 (89.8%), azithromycin 45 (91.8%)), ceftriaxone 47 (95.9%), chloramphenicol 46 (93.9%), erythromycin 46 (93.9%), penicillin 49 (100%), and vancomycin 45 (91.8%) (Table 3). Moreover, two (4.1%) of the 49 isolates were drug-resistant to two drugs, particularly amoxacillin, and eryomycin/vancomycin.

**Table 3:** Antimicrobial susceptibility patterns among group A *Streptococcus* isolates cultured from healthy school children—Jigjiga city, Eastern Ethiopia, April 12 to May 27, 2021

Antibiotics	Total isolates	Susceptible	Intermediate	Resistant
		**Number (%)**	**Number (%)**	**Number (%)**
Amoxicillin	49	44 (89.8%)	3(6.1%)	2(4.1)
Azithromycin	49	45 (91.8%)	4 (8.2%)	-
Ceftriaxone	49	47 (95.9%)	2 (4.1%)	-
Chloramphenicol	49	46 (93.9%)	3(6.1%)	-
Erythromycin	49	46 (93.9%)	2 (4.1%)	1(2.0%)
Penicillin	49	49 (100%)	-	-
Vancomyin	49	45 (91.8%)	4(8.2%)	-

## Discussion

In the present study the overall pharyngeal carriage rate of GAS was 10.6%. (95% CI; 8.1%—13.7%). A carrier rate comparable to ours has been recorded in Ethiopia (9.7% and 12.2%, respectively) [14, 15], United Arab Emirates (10%) [21], Nepal (10.8%) [22], and Yemen (12.8%) [23].

In contrast, the prevalence was lower than those reported in Uganda (16.0%) [24], Egypt (16%) [25], India (23.1%) [17], Argentina (14.2%) [26] and Brazil (14.0%) [27]. Our results showed a higher carriage rate than Gabon's 5.8% [27] and Nepal's 5.0% [28]. The possible explanation for the variation might be due hygiene, sample size, seasonal change, and geography, and socio-demographic [15, 23–25].

Children who lived with a family member who had a sore throat were twice as likely to colonize group A *Streptococcus* (GAS) than children who did not have a primary case or sore throat in their household. In Melbourne, Australia, researchers discovered that when a primary case is present, the likelihood of subsequent infection within a family increases by 1.8 times [29]. In addition, according to a study conducted in Nepal, one out of every five children is affected when a family member has already been infected [22].

Children from families with more than eleven members had a four-fold greater chance of carrying GAS than children from families with fewer members. Similarly, research in Hawassa found that children living in families with more than five members were more than 10 times more likely to be carriers of GAS than children living in families with fewer members. [15]. According to another study from Iraq, the carrier rate among children living in homes with more than six individuals is two times higher than among children living in families with fewer members [30]. According to the findings of these investigations, there is a substantial link between the carrier rate and the number of family members. The explanation for this could be crowding simply increases risk of disease (and colonization) transmission. Unlike a study by Asrat Anja [15], in this study Children living with non-immediate families were more than three times more likely to have GAS than children living with their immediate family. Crowding and poor hygiene, therefore, increases the chance of the transmission of GAS [31].

All GAS isolates were sensitive to penicillin in our study. The same high activity of penicillin had been reported in many countries, namely Ethiopia [14, 15, 32], Uganda [24], India [17], Nepal [22, 28] and Argentina [26]. Our findings reveal that amoxicillin (89.8%) and ceftriaxone (95.9%) have slightly reduced sensitivity against GAS when compared to recently published publications in Senegal and Ethiopia [15, 33]. For patients allergic to penicillin, erythromycin and other macrolides were suggested as first-line alternatives [6]*.* The isolates displayed the same level of sensitivity to erythromycin and azithromycin (93.9%). The isolates also showed 93.9% and 91.8% sensitivity to chloramphenicol and vancomycin, respectively. A comparable result was reported from Ethiopia [14, 15, 32] and other parts of the world [17, 24–26, 33, 34]. About 2(4.1%) of isolated GAS isolates were showed multiple drug resistant. A comparable result was reported from Hawwasa, Ethiopia [15].

### Limitation of the study

Since the study was a cross-sectional study conducted over a short period, the impact of environmental or seasonal factors on variations in prevalence could not be determined. Some of the children identified as being colonized could have been ill (not colonized). In addition, this study was unable to performed ASO titer due to a lack of resources.

## Conclusion

The present study showed a significant throat carriage of GAS in the Jigjiga city school children population. Children with a sore throat in the family, children from a large family, and children from non-immediate families have all been identified as independent predictors of the throat culture positive for GAS. The vast majority of GAS isolates cultured from the Jigjiga city school children were found to be sensitive to Penicillin, amoxicillin, ceftriaxone, chloramphenicol, erythromycin, azithromycin, and vancomycin.

The present study provides useful baseline information on the GAS carriage rate and resistance trend among healthy school children in this study area. Further study is important to understand the epidemiological features and to give the therapeutic strategies for public health problems due to antibiotic resistance.

## Data Availability

The data sets generated during and/or analyzed during the current study are available from the corresponding authors on reasonable request.

## Abbreviations

AOR: Adjusted Odds Ratio
ASO: Antistreptolysin O
AST: Antibiotic Susceptibility Test
CLSI: Clinical Laboratory Standard Institute
COR: Crude Odds Ratio
GAS: Group A *Streptococcus*
MHA: Mueller Hinton Agar
